# Hyperthyroidism is a Risk Factor for Developing Adhesive Capsulitis of the Shoulder: A Nationwide Longitudinal Population-Based Study

**DOI:** 10.1038/srep04183

**Published:** 2014-02-25

**Authors:** Shih-Wei Huang, Jia-Wei Lin, Wei-Te Wang, Chin-Wen Wu, Tsan-Hon Liou, Hui-Wen Lin

**Affiliations:** 1 Department of Physical Medicine and Rehabilitation, Shuang Ho Hospital, Taipei Medical University, Taipei, Taiwan; 2 Department of Neurosurgery, Shuang Ho Hospital, Taipei Medical University, Taipei, Taiwan; 3 Department of Physical Medicine and Rehabilitation, Changhua Christian Hospital, Changhua, Taiwan; 4 Graduate Institute of Injury Prevention, Taipei Medical University, Taipei, Taiwan; 5 Department of Mathematics, Soochow University, Taipei, Taiwan; 6 Evidence-Based Medicine Center, Wan Fang Hospital, Taipei Medical University, Taipei, Taiwan

## Abstract

The purpose of this study was to investigate the prevalence and risk of adhesive capsulitis among hyperthyroidism patients. The data were obtained from the Longitudinal Health Insurance Database 2005 (LHID 2005) in Taiwan, using 1 million participants and a prospective population-based 7-year cohort study of survival analysis. The ambulatory-care claim records of patients diagnosed according to the International Classification of Diseases, Ninth Revision, Clinical Modification (ICD-9-CM) codes relating to hyperthyroidism between January 1, 2004 and December 31, 2007, were obtained. The prevalence and the adjusted hazard ratio (HR) of adhesive capsulitis among hyperthyroid patients and the control group were estimated. Of 4472 hyperthyroid patients, 162 (671/100 000 person-years) experienced adhesive capsulitis during the 24 122 person-year follow-up period. The crude HR of stroke was 1.26 (95% confidence interval [CI], 1.06 to 1.49), which was larger than that of the control group. The adjusted HR of developing adhesive capsulitis was 1.22 (95% CI, 1.03 to 1.45) for hyperthyroid patients during the 7-year follow-up period, which achieved statistical significance. The results of our large-scale longitudinal population-based study indicated that hyperthyroidism is an independent risk factor of developing adhesive capsulitis.

Adhesive capsulitis of the shoulder is a condition characterized by intense shoulder pain, gradual fibrosis of the glenohumeral joint that causes a limited range of motion, and contracture of the glenohumeral joint capsule^1^. The prevalence of adhesive capsulitis is between 2% and 5%, and it primarily occurs in women. The incidence of adhesive capsulitis is between 2 to 3 percent in the general population, and it primarily occurs in women^2^. Disability because of adhesive capsulitis symptoms can influence work performance and increase public-health medical expenditures^3^. Pathogenesis mechanisms have been proposed, such as endocrine, immunological, and inflammatory processes^4^. In addition, thyroid diseases, diabetes mellitus, Dupuytren contractures, breast cancer treatment, and autoimmune diseases have been associated with adhesive capsulitis in previous studies^5^,^6^,^7^,^8^,^9^. Furthermore, patients diagnosed with myocardial infarctions and cerebral vascular diseases are reportedly at risk of adhesive capsulitis^10^,^11^.

A previous cross-sectional study reported that thyroid disorders are frequently accompanied by musculoskeletal disorders, such as adhesive capsulitis, Dupuytren contractures, trigger finger syndrome, and carpal tunnel syndrome. Overall, 137 patients with thyroid disorders were enrolled; of these, 15 had been diagnosed with adhesive capsulitis^12^. One case report described a subclinical hyperthyroid patient who was diagnosed with comorbid bilateral adhesive capsulitis^7^. The researchers stated that autonomic nervous system dysfunctions could play a key role in pathogenesis. The activation of the sympathetic nervous system in hyperthyroid patients may underlie the association of adhesive capsulitis and shoulder-hand syndrome^7^. However, the definitive pathogenesis factor of adhesive capsulitis in hyperthyroid patients remains undetermined.

Only one cross-sectional prevalence study on adhesive capsulitis in thyroid disorder patients has been conducted^12^. The influence of thyroid disorder status on developing adhesive capsulitis was not determined in this study, and longitudinal and large-scale studies are lacking. Limited information is available regarding the risk of adhesive capsulitis in hyperthyroid patients. Therefore, we conducted a longitudinal population-based study, controlling risk factors such as diabetes and dyslipidemia, to investigate the risk of hyperthyroid patients developing adhesive capsulitis.

## Results

**Figure 1:**
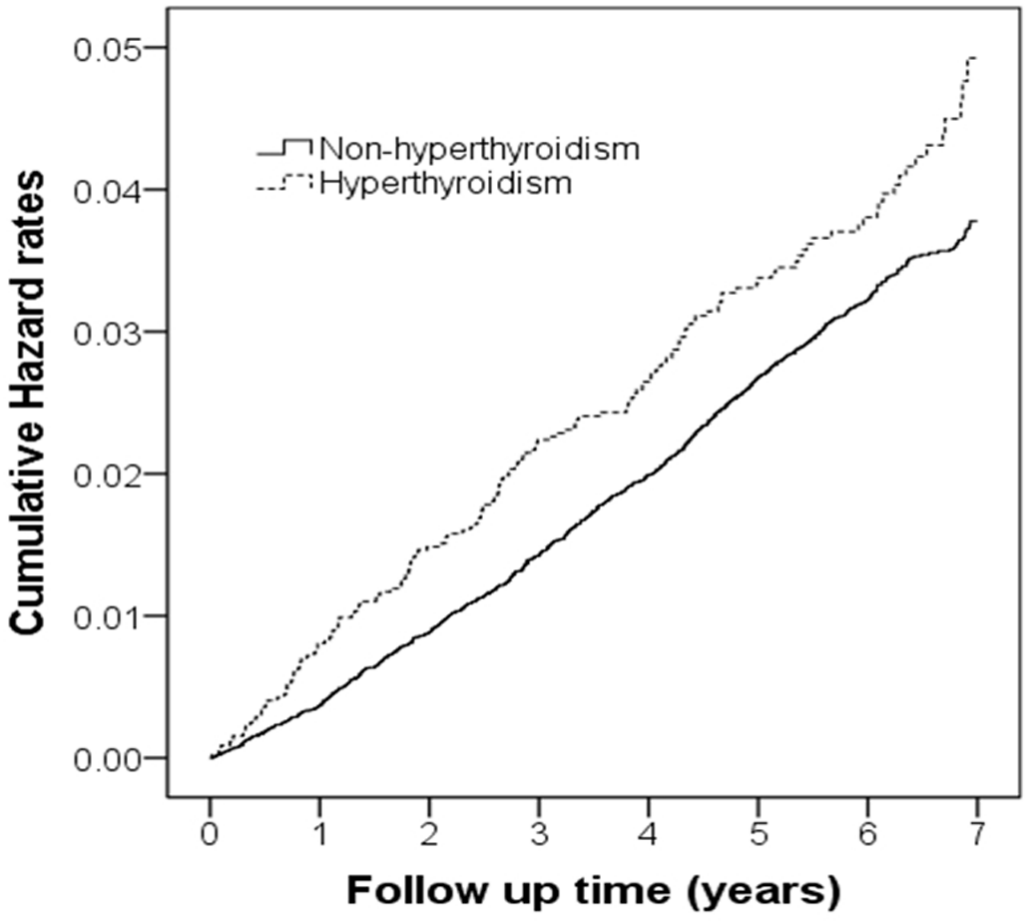
A hazard rate plot constructed using the Kaplan-Meier method of hyperthyroid patients with frozen shoulders and the control group for 7 y. Log-rank test *P* = .007.

We identified 4472 hyperthyroid patients in the age- and sex-matched control cohorts. Compared with the control cohort (Table 1), hyperthyroid patients are more likely to have comorbid autoimmune diseases (*P* < .001), diabetes mellitus (*P* < .001), hypertension (*P* < .001), hyperlipidemia (*P* < .001), coronary heart disease (*P* < .001), and chronic liver disease (*P* < .001). Figure 1 presents the hazard curves of hyperthyroid patients diagnosed with adhesive capsulitis and the control cohort during the 7-year follow-up period, after adjusting for patient age, sex, diabetes mellitus, coronary heart disease, hypertension, hyperlipidemia, autoimmune diseases, chronic liver disease, cancer, and urbanization level (log-rank test, *P* < .001).

Table 2 shows the incidence and hazard ratios (HRs) of adhesives capsulitis in hyperthyroid patients and the control cohort. Of the 4472 hyperthyroid patients, 162 patients (671/100 000 person-years) exhibited adhesive capsulitis during the 24 122 person-year follow-up period. The crude HR of stroke was 1.26 (95% confidence interval [CI], 1.06 to 1.49), which was larger than that obtained for the control group.

Table 3 presents the adjusted HRs of adhesive capsulitis in both the hyperthyroid patients and control cohort. The adjusted HR obtained for hyperthyroid patients was 1.22 (95% CI, 1.03 to 1.45), which was statistically significant. In addition, other confounding factors of adhesive capsulitis were analyzed. The results showed that age (adjusted HR: 1.05, 95% CI, 1.04 to 1.05), the male sex (adjusted HR: 069, 95% CI, 0.58 to 0.82), hyperlipidemia (adjusted HR: 1.49, 95% CI, 1.25 to 1.77), and chronic liver disease (adjusted HR: 1.37, 95% CI, 1.14 to 1.66) were all statistically significant regarding the development of frozen shoulder during the 7-year follow-up period.

## Discussion

This study showed that hyperthyroid patients have 1.22 times the risk of developing adhesive capsulitis compared to the general population. Until now, no relevant large-scale longitudinal population-based study has been conducted on the risk of adhesive capsulitis in hyperthyroid patients. All previously conducted studies regarding the risk of adhesive capsulitis in thyroid-disorder patients have been small-sample cross-sectional studies^12^. Limited information has been obtained regarding the temporal relationship between hyperthyroidism and adhesive capsulitis in previous studies. We adjusted for factors, such as diabetes and dyslipidemia, and identified hyperthyroid patients who were subsequently diagnosed with adhesive capsulitis. The results show that hyperthyroidism patients have a high comorbidity risk of adhesive capsulitis.

The definite pathogenesis of adhesive capsulitis remains under investigation. Shoulder pain onset that indicates adhesive capsulitis is considered to be mediated through nerve stimulation. Adhesive-capsulitis shoulder pain can be caused by alpha-adrenoreceptor hyperresponsiveness, in which both nociceptive and proprioceptive fibers are stimulated, resulting in pain^13^. The inflammatory process is considered another pathogenesis of adhesive capsulitis. Rodeo et al found elevated levels of pro-inflammatory cytokines in adhesive capsulitis patients^14^. They proposed that the stimulation of inflammation caused by cytokines can engender shoulder synovitis, and this can result in a fibrotic cascade that is associated with growth factors such as TGF-beta^14^. The chronic fibrosis process of capsulitis is confirmed by observing the histologic presentation of fibroblast cell proliferation. Matrix metalloproteinase and fibrogenic growth factors are also increased in adhesive capsulitis patients^15^.

Similar to the inflammatory and fibrosis process pathogenesis of adhesive capsulitis patients, hyperthyroid patients also present an inflammatory cytokine release and fibrosis phenomenon. Regarding thyroid disorder-associated ophthalmopathy patients, cell-mediated (Th1) and humoral-mediated immune responses infiltrate the orbital area. High levels of IL-2, IFN-γ, and TNF-α cytokines are secreted by Th1 cells in the retro-orbital area of patients who are diagnosed with Graves disease^16^,^17^. However, another study found Th2 cell-secreted cytokines, such as IFN-γ, IL-4, and IL-10, in patients^18^. Moreover, a previous study described the cytokine profiles of patients diagnosed with Graves disease and thyroid disorder-associated ophthalmopathy^17^. Cytokines have been proven as capable of inducing several proteins in orbital fibroblasts, and these cytokines have the ability to stimulate orbital fibroblast proliferation^19^. We propose that cytokine and fibroblast proliferation contributes to not only the process of thyroid ophthalmopathy but also to adhesive capsulitis. This can explain why hyperthyroid patients are vulnerable to adhesive capsulitis.

SLE and RA patients were analyzed in this study, but were not found to have a high risk of adhesive capsulitis. Regarding SLE patients, cytokines have been proposed to have a pathogenic role in autoantibody production and immune complex deposition. These cytokines are interleukin-6, interleukin-17, interleukin-18, type I interferons, and TNF-alpha^20^. Cytokines have also been found in the pathogenesis of rheumatoid arthritis. One review article provided a comprehensive list of related cytokines, such as TNF-alpha, IL 1, 6, 15, 17, and 18, GM-CSF, VEGF, and TGF-beta^21^. However, this article stated that how these cytokines are organized within a hierarchical regulatory network remains unclear. In addition, the article stated that TNF-alpha plays a key role because TNF-alpha-blockage agents can are involved in successfully treating RA^21^. We propose that the pro-inflammatory cytokine and fibrosis process modulation is different in these autoimmune diseases and adhesive capsulitis.

We controlled other possible hazard factors for developing adhesive capsulitis. In addition to hyperthyroidism, the results showed that hyperlipidemia patients have a high risk of adhesive capsulitis. These results support those of a previous case-controlled study that analyzed the lipid profiles of patients diagnosed with frozen shoulders and found that fasting serum triglyceride and cholesterol levels were increased in these patients compared with the levels of participants without frozen shoulders^22^. However, the pathogenesis of adhesive capsulitis in hyperlipidemia patients remains unclear.

In contrast to a previous population-based longitudinal study, our results showed that diabetes mellitus patients do not have an increased risk of adhesive capsulitis^23^. This could be because the controlled group selection was different from that of the previous study either because of differing circumstances or participant matching to the hyperthyroid group. Our patients were predominantly women and were younger than those in the previous study on diabetes mellitus and frozen shoulders. A different control group selection can result in the influence of confounding factors. Long longitudinal follow-up data collection is the strength of our study. In addition, we attempted to control confounding factors, such as diabetes, hyperlipidemia, and autoimmune diseases such as SLE and RA. The long follow-up period and large number of potential confounding factors that were considered in this study enabled producing reliable results.

The LHID2005 data released by the Taiwan NHI Institutes were used in this study. However, this study has several possible limitations. First, the diagnosis of adhesive capsulitis and hyperthyroidism was determined using the ICD codes listed in the NHI claim database; however, the diagnostic accuracy of the results obtained from the database were not confirmed. To increase the accuracy of diagnoses, the NHI Bureau has formed various audit committees that randomly sample claim data regularly to verify diagnostic validity and care quality. In addition, we used only consecutively coded cases to avoid inaccurate codes in the database records. These methods might improve the accuracy of registering rheumatic diseases. Second, the NHIRD records do not contain the laboratory data of hyperthyroid patients, and information regarding disease stratification severity is also limited. Third, the influences of thyroid medications, I131 radioiodine therapy, and thyroid surgery were not analyzed. Finally, our results were based on a retrospective cohort study. Information regarding lifestyles, obesity, cigarette smoking, and alcohol consumption cannot be obtained from the administrative database.

## Conclusion

In conclusion, our 7-year longitudinal population-based case-controlled cohort study results showed that hyperthyroid patients have a 1.22-fold higher risk of adhesive capsulitis than that of patients without this condition. In addition to hyperthyroidism, the results showed that hyperlipidemia is another risk factor of adhesive capsulitis. Additional studies regarding the influence of hyperthyroidism treatment on the risk of adhesive capsulitis are recommended.

## Methods

We analyzed records that were obtained from the Longitudinal Health Insurance Database 2005 (LHID2005), which is maintained by the Taiwan National Health Insurance (NHI) Institutes. The LHID contains data of all health insurance claims, including demographic, inpatient care, ambulatory care, and prescription drug data, as well as International Classification of Diseases, Ninth Revision, Clinical Modification diagnostic codes. Overall, the records of 1 000 000 beneficiaries, who were enrolled in 2005, were randomly sampled from the records of the 25.56 million people contained in this database (the NHI program insures approximately 99% of the population in Taiwan). To protect the privacy of personal data, the records obtained from the database were de-identified. In addition, informed consent was waived in this secondary data analysis study.

The study cohort contained all patients who had been diagnosed with a hyperthyroid disease (ICD-9-CM codes 242.9) between January 1, 2004 and December 31, 2007 according to data on ambulatory medical care visits. The hyperthyroid group consisted of patients who had received a principal diagnosis of hyperthyroidism (ICD-9-CM codes 242.9) during an ambulatory medical care visit between January 1, 2004 and December 31, 2007. To improve the diagnosis accuracy, only patients who had at least 2 consecutive ambulatory visits in which the principal diagnosis was hyperthyroidism were recruited in this study (N = 4779). Patients who were diagnosed with adhesive capsulitis (ICD-9-CM codes 726.0) prior to a thyroid disorder diagnosis (N = 57, hyperthyroidism) or multiple diagnosed with other thyroid disease such as thyroid cancer or hypothyroidism (N = 85) or whose records were missing variables, such as date of birth, sex, and age < 18 years (N = 165) were excluded. A total of 4472 hyperthyroid patients met the inclusion and exclusion criteria and were enrolled in this study. Patients in the control group were matched with those in the study cohort (5 control patients per case patient) according to age (< = 30, 31–40, 41–50, 51–60, 61–70, and >70 y) and sex by using the remaining patient records obtained from the LHID. Patients were excluded if they had been diagnosed with a thyroid disorder between 2004 and 2010, or had been diagnosed with adhesive capsulitis before 2004.

### Confounders and baseline variables

We obtained baseline variables, including age, sex, urbanization level (stratified into 3 levels: urban, suburban and rural), and confounding factors, such as diabetes mellitus (DM; ICD-9-CM code 250), hypertension (ICD-9-CM codes 401–405), hyperlipidemia (ICD-9-CM codes 272.0–272.4), autoimmune diseases (rheumatoid arthritis: ICD-9-CM code 714.0; SLE ICD-9-CM code 710.0), chronic liver disease (ICD-9-CM code 571), cancer (ICD-9-CM codes 140-208), and coronary heart disease (ICD-9-CM codes 410–414), for all patients.

### Outcome measures

We used the initial diagnosis of adhesive capsulitis (ICD-9-CM codes 726.0) as the study endpoint. All participants were followed from the index date until the occurrence of the endpoint or until December 31, 2010, whichever was first, and final-date observations were censored observations.

### Statistical analysis

The Pearson chi-squared test or the Fisher exact test was applied to compare demographic characteristics and comorbidities. The Cox proportional hazard model was used to evaluate the hazard rates of adhesive capsulitis between the study and control cohorts, after adjusting for potential confounding factors, constituting patient age (as continuous), sex, diabetes mellitus, coronary heart disease, hypertension, hyperlipidemia, urbanization level, autoimmune disease, chronic liver disease, and cancer. To fulfill the proportional-hazard assumption, exploratory diagnostic log-log survival plots were applied to verify the proportionality of each dichotomous variable in the model and meet the proportional-hazard assumption. We plotted the stroke hazard curves based on the Cox model of the patient and control cohorts after adjusting for potential confounding factors. The SAS statistical package (SAS System for Windows, Version 9.1.3, SAS Institute Inc., Cary, NC, USA) and SPSS version 20 were used for analysis. A *P* value < .05 was considered statistically significant.

## Author Contributions

Conceived and designed the experiments: S.W.H., J.W.L. Performed the experiments: S.W.H., H.W.L. Analyzed the data: H.W.L., W.T.W. Prepare Tables and Figure: T.H.L., C.W.W. Wrote the paper: S.W.H. All authors reviewed the manuscript.

## Figures and Tables

**Table 1:** Baseline variables including demographic characteristics and comorbid medical disorders of the 2 age- and sex-matched cohorts (*N* = 26 832)

	Hyperthyroid patients*n* = 4472		control patients*n* = 22360		
Baseline Variable	No.	(%)	No.	(%)	*P* value
**Characteristics**					
Age (y)					
18–30	1114	24.9	5570	24.9	
31–40	1207	27.0	6035	27.0	
41–50	1066	23.8	5330	23.8	
51–60	645	14.4	3225	14.4	
61–70	287	6.4	1435	6.4	
>70	153	3.4	765	3.4	
Sex					
Male	989	22.1	4945	22.1	
Female	3483	77.9	17 415	77.9	
Urbanization level					.076
Urban	2824	63.1	13 788	61.7	
Suburban	1206	27.0	6405	28.6	
Rural	442	9.9	2167	9.7	
**Comorbid medical disorders**					
Autoimmune disease					<.001
Yes	145	3.2	412	1.8	
No	4327	96.8	21 948	98.2	
Diabetes mellitus					<.001
Yes	360	8.1	1435	6.4	
No	4112	91.9	20 925	93.6	
Hypertension					<.001
Yes	716	16.0	3060	13.7	
No	3756	84.0	19 300	86.3	
Hyperlipidemia					<.001
Yes	551	12.3	2114	9.5	
No	3921	87.7	20 246	90.5	
Coronary heart disease					<.001
Yes	327	7.3	1136	5.1	
No	4145	92.7	21 224	94.9	
Chronic liver disease					<.001
Yes	601	13.4	1850	8.3	
No	3871	86.6	20 510	91.7	
Cancer					.005
Yes	134	3.0	509	2.3	
No	4338	97.0	21 851	97.7	

**Table 2:** Incidence of frozen shoulder among hyperthyroid patients during the 7-year follow-up period

Presence of frozen shoulder	Controls	Hyperthyroid patients
follow-up period		
Yes/Total	740/22 360	162/4472
person-years	137 485	24 122
Incidence per 100 000 person-years	538	671
Crude HR (95% CI)	1.00	1.26 (1.06–1.49)

**Table 3:** Adjusted hazard ratios and 95% confidence intervals of frozen shoulders among hyperthyroid patients during the 7-y follow-up period

	Presence of frozen shoulder		
Variable	Adjusted Hazard Ratio	95% CI	*P* value
Hyperthyroidism	1.22	1.03–1.45	.019
Age (y)	1.05	1.04–1.05	<.001
Sex (male)	0.69	0.58–0.82	<.001
Urbanization level			
Rural	1.00		
Suburban	0.90	0.77–1.04	.161
Urban	0.90	0.72–1.11	.336
Diabetes mellitus	1.05	0.86–1.29	.593
Autoimmune disease	0.99	0.67–1.45	.956
Hypertension	1.01	0.85–1.20	.881
Hyperlipidemia	1.49	1.25–1.77	<.001
Coronary heart disease	1.03	0.83–1.28	.774
Chronic liver disease	1.37	1.14–1.66	.001
Cancer	0.82	0.57–1.18	.289
